# Decreases in influenza vaccination coverage among nursing home healthcare workers and in measures to promote influenza vaccination, France, 2007/08 to 2024/25

**DOI:** 10.2807/1560-7917.ES.2026.31.12.2500628

**Published:** 2026-03-26

**Authors:** Remi Hanguehard, Laure Fonteneau, Nathalie Bonnet, Daniel Levy-Bruhl, Isabelle Parent-du-Châtelet, Sophie Vaux

**Affiliations:** 1 Santé publique France, Saint-Maurice, France

**Keywords:** vaccination, influenza, nursing homes, healthcare workers, vaccination coverage, COVID-19 pandemic

## Abstract

**BACKGROUND:**

Vaccination of residents and healthcare workers (HCWs) against influenza in nursing homes is an important prevention strategy.

**AIM:**

To describe trends in influenza vaccination coverage (VC) among HCWs working in nursing homes in France and measures implemented to support vaccination campaigns from 2007 to 2025, and to identify effectiveness of these measures.

**METHODS:**

We analysed data from seven nationwide cross-sectional studies conducted between seasons 2007/08 and 2024/25 and performed multivariate analysis using negative binomial regressions, to identify determinants.

**RESULTS:**

National influenza VC among HCWs decreased from 37.2% (95% CI: 35.7–39.4) in 2007/08 to 24.2% (95% CI: 23.2–25.1) in 2024/25 (mean: 39,740 HCWs per season, 6 seasons). Vaccination coverage disparities by category of HCWs (2024/25 VC of physicians: 56.3%; nurses: 34.2%; nursing assistants: 19.3%) were observed throughout the period. Measures to promote influenza vaccination were less frequently implemented over time: support of vaccination campaigns by the management team (from 2018/19: 89% to 2024/25: 52%), implementation of collective (68% to 49%) or individual information sessions (19% to 9%), distribution of information on influenza vaccines (64% to 53%) or influenza (83% to 69%). Seven measures exhibited effectiveness in 2018/19 compared to only four in 2024/25, which had less effectiveness. Management teams highlighted the strong reluctance of HCWs to receive influenza vaccination.

**CONCLUSIONS:**

Influenza VC among HCWs in nursing homes is marked by disparities among professional categories. To mitigate the decline in VC among HCWs, it is essential to implement effective measures to support the campaigns.

**Key public health message**

**What did you want to address in this study and why?**

Vaccinating healthcare workers (HCWs) in nursing homes can potentially reduce the overall number of influenza cases and mortality of at-risk residents. We studied how influenza vaccination coverage (VC) among HCWs changed from 2007 to 2025, which actions were taken to promote vaccination among HCWs over this period and how effective these actions were. This knowledge helps improve future vaccination campaigns.

**What have we learnt from this study?**

Since the COVID-19 pandemic, VC of HCWs working in nursing homes for older residents (aged > 60 years) in France has declined. Influenza VC decreased in all professional HCW categories over the period (2018/19 to 2024/25): VC of all HCWs decreased from 37% to 24%; physicians: from 76% to 56%, nurses: from 43% to 34%, nursing assistants: from 27% to 19%.

**What are the implications of your findings for public health?**

A remarkable decline in the implementation of measures to promote HCW’s influenza vaccination has been observed between 2018/19 and 2024/25. Management teams in nursing home highlight strong reluctance of healthcare workers to influenza vaccination. It is essential to implement effective measures to support the seasonal campaigns among HCWs.

## Introduction

Influenza virus infection is a major public health challenge, given the high morbidity and mortality, especially in older individuals [1,2]. Vaccination against influenza for nursing home (NH) residents has proven effective in preventing respiratory illness, hospitalisation and death [3]. However, influenza outbreaks in NHs occur often, despite high influenza vaccination coverage (VC) rates among residents [4,5]. During the 2024/25 influenza season in France, 4,071 clusters of acute respiratory infections cases were reported by NHs for elderly individuals (aged > 60 years), with 1,955 of these clusters attributed to influenza, i.e. 53% of those tested for a specific pathogen [6]. 

Vaccinating healthcare workers (HCWs) in NHs can potentially contribute to the prevention of influenza cases, reducing the overall number of influenza virus infections within the NHs and mortality of at-risk residents. However, proof of the real-life effectiveness of vaccinating HCWs to indirectly protect residents is difficult to provide. Although some studies suggest a protective effect, the evidence is not very strong [7,8]. 

Currently, French recommendations for yearly seasonal influenza vaccination include all individuals aged over 64 years, residents of NHs and professionals who are in contact with NH residents; the objective is to achieve 75% VC among HCW and professionals in contact with residents at risk [9]. Despite recommendations, influenza VC of HCWs remained low (37% in 2008, 32% in 2018) in France [10,11], as in other countries [12]. The emergence of SARS-CoV-2 in 2020 and the ensuing COVID-19 pandemic may have had an impact on influenza VC of HCWs working in NHs as well as on the measures taken in NHs to support vaccination campaign against influenza for HCWs.

The aims of our study were (i) to describe trends in influenza VC among HCW working in NHs in France between 2007/08 and 2024/25 (6 seasons), taking into account HCW categories (physicians, nurses, nursing assistants and other paramedical personnel), (ii) to describe the measures implemented to support influenza vaccination campaigns for HCW from 2007/08 to 2024/25 (7 seasons) and (iii) to evaluate their effectiveness during 2018/19, 2023/24 and 2024/25 (3 seasons).

## Methods

### Study design

In order to estimate influenza VC among HCW working in NHs, we conducted seven nationwide VC studies in France during the following seasons of vaccination campaigns: 2007/08, 2018/19, 2020/21, 2021/22, 2022/23, 2023/24 and 2024/25. These studies were conducted in metropolitan France and overseas territories, i.e. Guadeloupe and Martinique (French Caribbean), French Guiana (South America), Reunion Island (Indian Ocean), with the exception of the 2007/08 VC study, which was conducted only in metropolitan France. In metropolitan France, in Guadeloupe, Martinique and French Guyana, vaccination campaigns are organised from mid-October to the end of January and sometimes extended until the end of February, depending on the epidemiological situation. On Réunion Island, located in the Southern Hemisphere, vaccination campaigns are generally organised from mid-May to the end of September. Nursing homes consider vaccinations administered during the previous austral winter. The VC studies started after the campaign for each season concluded. 

Nursing homes were invited to participate by email (or post, only 2007/08) by Santé publique France and reminders to participate were sent by the regional healthcare-associated infection control centre (Cpias: https://www.cpias.fr) or the regional health agency (ARS: https://www.ars.sante.fr). At least one reminder was sent 3 weeks after the initial solicitation. Data collection was conducted from February to early April. During the 2020/21 season, because of the COVID-19 pandemic, the VC study was implemented in January 2021 at the request of the Ministry of Health on account of a shortage of influenza vaccines. For this specific season, the vaccination campaign was not completed, and participation from NHs was very low.

### Nursing home selection

For the 2007/08 and 2018/19 seasons, we performed a cross-sectional study of NHs using a single stage stratified random sampling design. The sampling frames were the list of NHs recorded in France’s national medico-social and healthcare institution database (FINESS, https://www.sae-diffusion.sante.gouv.fr/sae-diffusion/accueil.htm). The methodologies used in these studies have already been described [10,11].

For the five annual VC studies from 2020/21 to 2024/25, all French NHs were invited to participate, with data from 2020/21 incomplete.

### Study population

The study population consisted of salaried HCWs including physicians, nurses, nursing assistants and other paramedical personnel (not included in 2007/08). Healthcare workers employed for less than 3 months and students (medical or nursing students) were not included in the study.

The NHs included in the VC studies were medico-social establishments providing accommodation for dependent elderly people (aged > 60 years), where residents stay overnight. Short-stay care facilities and retirement homes with individual apartments were not included.

### Data collection

For each NH, only aggregated data were collected. A dedicated questionnaire (Supplement S1 provides questionnaire of the 2024/25 VC study) was completed by a member of the management team: the coordinating physician, the director or the nursing care coordinator. They were asked about vaccinations for the season that just ended. Data were collected using a postal questionnaire during the 2007/08 season and online questionnaires for the following seasons (built using Voozanoo 3 or 4: https://www.epiconcept.fr/projets-sur-mesure/socle-technique-voozanoo-epicraft). We made minimal changes to the questionnaires between seasons to ensure comparability.

Data on NH administrative and structural characteristics were collected directly from the NHs or by merging with administrative databases: status (public, private, private non-profit), region, size (number of beds) and affiliation to a hospital. To estimate VC, we collected two types of data: (i) number of HCWs per occupational category during the season (denominators used to calculate VC) and (ii) number of HCWs vaccinated against influenza per occupational category during the vaccination campaign (numerators used to calculate VC). HCWs were included regardless of whether they were vaccinated inside or outside the NH.

We also asked the management team to report the measures implemented in the NH to support the influenza vaccination campaign during the season (see Supplement S1: Questionnaire). Multiple answers were possible: organisation of vaccination for professionals by the occupational health service, vaccination free of charge for HCWs in the NH, influenza vaccination promotion for HCWs with use of informative posters, use of educational videos or games, type of information disseminated, information about influenza or influenza vaccines, organisation of individual or collective information sessions, nomination of a point of contact for vaccination in the NH (defined as a medical or paramedical HCW who could provide reliable information on influenza vaccination), existence of an in-house multidisciplinary group on vaccination, involvement of the management team (NH director, physician care coordinator or nursing care coordinator) to support the influenza vaccination campaign (though email, meetings, direct involvement) or other measures (free text space). In 2007/08, only information on the following measures was collected: free vaccination for HCWs in NH and the organisation of collective information sessions. In 2024/25, the NHs that had implemented few measures in total (no minimum threshold for the number of measures was defined, and based on the perspective of the management team at the NH) or fewer measures compared to previous years were asked for a reason why (multiple-choice, closed-ended). Nursing homes had the choice to respond to only part of the questionnaire, if preferred.

### Data analysis

The descriptive analyses of VC were performed according to HCW occupational category (physicians, nurses, nursing assistants and other paramedical personnel) for each season. We dichotomised NH size into 1–99 beds and ≥ 100 beds.

Outcomes were given in percentages with 95% confidence intervals (CI). The trends in VC and implemented actions compared the seasons that followed the emergence of COVID-19 (2021/22, 2022/23, 2023/24 and 2024/25) with the last available pre-COVID-19 pandemic season (2018/19). Percentages were compared between seasons using the Fisher’s exact test.

In order to assess the determinants of influenza VC and measures that could improve influenza VC among HCWs in NHs during the 2018/19, 2023/24 and 2024/25 seasons, and acknowledging data aggregation and overdispersion, we performed univariate and multivariate analyses using negative binomial regressions. The determinants tested were the administrative characteristics described above, as well as the various measures implemented in the NH to support the influenza vaccination campaign. Adjusted prevalence ratios (PRa) and their 95% confidence intervals were used as measures of association with VC. All determinants with a p value < 0.2 in the univariate analysis were introduced in the multivariate model. A p value ≤ 0.05 was considered statistically significant. Non-significant variables were removed one by one (backward elimination). Only significant variables were kept in the final multivariate analysis. To assess whether any of the variables in the final model were subject to confounding by variables omitted from the model, each omitted variable was re-introduced individually.

We estimated VC in NHs that implemented several measures identified as effective in our multivariate analyses in 2018/19, 2023/24 and 2024/25, as well as in those that did not implement any effective measure. For the 2020/21 season, because of low participation of NHs, VCs of the HCW categories were not estimated, and were presented separately.

Data analyses were performed using Stata 14.2 and Stata 18 in 2023/24 and 2024/25 (StataCorp).

## Results

Depending on the season, the number of NHs participating in the studies ranged from 589 (2018/19) to 2,630 (2024/25). The VC studies included data on 20,420 HCWs (2018/19) and 58,852 HCWs (2024/25) (Table 1). Supplementary Table S2 provides the numbers of NHs and participation of NHs included in the study by region in France from 2007/08 to 2024/25 (6 seasons in total).

**Table 1:** Numbers of nursing homes and healthcare workers included in the study, France, 2007/08–2024/25 seasons^a^

Characteristics	Participation per season					
	2007/08	2018/19	2021/22	2022/23	2023/24	2024/25
**Numbers of nursing homes **						
Number invited to participate in the study^b^	2,186	1,189	7,492	7,457	7,424	7,420
Number included in the study^c^	1,218	589	1,431	2,006	2,370	2,630
Participation in the study (%)	55.7	49.5	19.1	26.9	31.9	35.4
Number included in the VC analyses^d^	1,218	558	1,091	1,796	2,070	2,292
Participation in VC analyses (%)	55.7	46.9	14.6	24.1	27.9	30.9
**Numbers of HCWs included in the VC estimates^e^**						
**Total**	**35,556**	**20,420**	**23,380**	**44,968**	**55,283**	**58,852**
Physicians	1,870	645	660	1,838	2,456	2,426
Nurses	8,174	3,506	4,125	8,317	10,512	10,556
Nursing assistants	25,512	13,948	17,401	32,308	39,492	42,885
Other paramedical personnel^f^	NA	2,321	1,194	2,505	2,823	2,985

HCW: healthcare worker; NA: not available; NH: nursing home; VC: vaccination coverage.

^a^ During the 2020/21 season, the number of participating nursing homes was very small (n = 256), the vaccination campaign for HCWs was completed in only 50 nursing homes. These data were excluded from the table.

^b^ The 2007/08 and 2018/19 were sample-based studies; during other seasons, all NHs were invited to participate.

^c^ Includes all NHs that participated in the study, whether for monitoring VC among HCWs (data from 2007/08 to 2024/25) or measures implemented to support influenza vaccination. The questionnaire is provided in Supplement S1).

^d^ Includes all NHs that responded to the questionnaire designed to monitor VC among HCWs and whose response quality allowed inclusion in the VC analyses.

^e^ Correspond to the denominators of the VC analyses.

^f^ Other paramedical personnel were not included for the 2007/08 season.

### Influenza vaccination coverage

When considering all HCWs combined, influenza VC decreased from 37.2% (95% CI: 35.7–39.4) in 2007/08, to 31.9% (29.7–34.1) in 2018/19 and 24.2% (23.2–25.1) in 2024/25. There was a significant decline over the four post-COVID-19 pandemic seasons (p < 0.001 for all seasons), compared with 2018/19 (Table 2, Supplementary Figure S3 provides a figure of the VC from 2007/08 to 2024/25). In January 2021, the first season following the emergence of SARS-CoV-2, the VC reached 55% but data on vaccination were reported by only 50 NHs.

**Table 2:** Influenza vaccination coverage of healthcare workers in nursing homes, France, 2007/08–2024/25 seasons^a^

Category	Influenza vaccination coverage															
	2007/08		2018/19		2021/22			2022/23			2023/24			2024/25		
	%	95% CI	%	95% CI	%	95% CI	p	%	95% CI	p	%	95% CI	p	%	95% CI	p
All healthcare workers^b^	37.2	35.7–39.4	31.9	29.7–34.1	30.0	28.3–31.6	0.001	28.1	26.8–29.5	0.001	25.7	24.7–26.6	0.001	24.2	23.2–25.1	0.001
Physicians	60.4	54.9–65.8	75.5	69.3–81.7	75.0	71.1–79.0	NS	62.4	50.2–74.6	0.001	60.1	53.2–67.0	0.001	56.3	48.5–64.2	0.001
Nurses	45.2	42.8–47.5	42.9	39.4–46.4	41.6	39.2–44.0	NS	37.5	34.8–40.2	0.001	35.5	33.9–37.1	0.001	34.2	32.6–35.8	0.001
Nursing assistants	33.7	31.8–35.6	26.7	24.5–29.0	24.6	23.0–26.2	0.001	23.3	22.2–24.5	0.001	20.3	19.4–21.3	0.001	19.3	18.4–20.2	0.001
Other paramedical personnel^c^	NA		34.0	30.1–38.0	36.0	32.5–39.5	NS	34.3	29.1–39.5	NS	32.5	30.0–35.1	NS	32.7	29.5–35.9	NS

CI: confidence interval; NA: not available; NS: non-significant.

The difference in VC between the season under consideration and the 2018/19 season (the most recent pre-COVID season for which estimates are available) was determined by Fisher’s exact test. No comparison was made to the 2007/08 season.

^a^ For the 2020/21 season, influenza VC for all healthcare workers was 55.2% (95% CI: 52.0–58.6). Vaccination of HCW was completed for only 50 nursing homes.

^b^ For more details on the number of healthcare workers included each year in each category, see Table 1.

^c^ Other paramedical personnel were not included for the 2007/08 season.

Different trends were observed depending on the HCW category. For physicians, influenza VC was 60.4% (95% CI: 54.9–65.8) in 2007/08, increased to 75% in 2018/19 and 2021/22, then decreased significantly in the following seasons to 56.3% (95% CI: 48.5–64.2) in 2024/25 with an absolute reduction of −19.2 in comparison with 2018/19. For nurses, the VC for influenza was 45.2% (95% CI: 42.8–47.5) in 2007/08. A downward trend was observed in 2018/19, followed by a statistically significant drop from 2022/23. Vaccination coverage for nurses fell by 8.7 points between 2018/19 and 2024/25. For nursing assistants, influenza VC steadily declined throughout the study period from 33.7% (95% CI: 31.8–35.6) in 2007/08 to 19.3% (95% CI: 18.4–20.2) in 2024/25. For other paramedical personnel, VC remained low and relatively stable across seasons (between 32.5 and 36.0) without significant changes.

Independent of season, influenza VC was significantly higher for physicians than for nurses and nursing assistants as observed by the non-overlapping of the confidence intervals (Table 2). The difference in VCs between physicians and nursing assistants was 27 points in 2007/08, rising to 49 points in 2018/19 and 37 points in 2024/25.

Supplementary Figure S4 provides regional VC data for the 2018/19 and 2024/25 seasons and links to national publications for other seasons.

### Measures implemented in nursing homes to promote influenza vaccination of healthcare workers

During the 2024/25 season, the offer of influenza vaccination free of charge for HCWs was implemented by 93% (95% CI: 92–94) of NHs, 82% (95% CI: 81–84) of NHs used posters for the promotion of the vaccination campaign, 69% (95% CI: 67–71) provided information about influenza to HCWs, 53% (95% CI: 51–55) provided information about influenza vaccines, 49% (95% CI: 47–51) organised collective information sessions, 9% (95% CI: 8–11) individual information sessions and 11% (95% CI: 10–13) used videos or games. The management team supported the vaccination campaign in 52% (95% CI: 50–54) of the NHs. A point of contact for vaccination was nominated within 30% (95% CI: 28–32) of the NHs (Table 3). Supplementary Figure S5 provides an overview of the measures implemented from 2007/08 to 2024/25.

**Table 3:** Measures implemented in nursing homes promoting influenza vaccination in healthcare workers, France, 2007/08–2024/25

Measures implemented^a^	Proportion of nursing homes implementing measures																
	2007/08		2018/19		2020/21	2021/22			2022/23			2023/24			2024/25		
	%	95% CI	%	95% CI	%	%	95% CI	p	%	95% CI	p	%	95% CI	p	%	95% CI	p
Free-of-charge vaccination for HCWs	90	88–92	94	92–96	NA	91	88–93	*	91	89–92	*	89	87–90	***	93	92–94	NS
Posters	NA		91	88–93	84	86	83–88	**	83	81–85	***	80	78–82	***	82	80–84	***
The management team support the campaign^b^	NA		89	86–92	88	55	51–59	***	57	55–60	***	53	50–55	***	52	50–54	***
Information about influenza	NA		83	80–86	82	78	75–82	*	66	63–69	***	64	63–66	***	69	67–71	***
Collective information sessions	49	46–52	68	63–71	65	48	44–52	***	39	37–42	***	45	43–47	***	49	47–51	***
Information about influenza vaccines	NA		64	59–68	75	50	47–54	***	50	47–52	***	48	46–50	***	53	51–55	***
Point of contact for vaccination nominated^c^	NA		33	29–37	37	16	13–19	***	24	22–26	***	31	29–34	NS	30	28–32	NS
Individual information sessions	NA		19	16–23	16	9	7–12	***	9	8–11	***	8	9–11	***	9	8–11	***
Videos or games	NA		8	6 – 11	16	20	17–24	***	9	7–10	NS	8	7–9	NS	11	10–13	NS

CI: confidence interval; HCW: healthcare worker; NA: not available; NS: non-significant.

^a^ Measures are presented by decreasing frequency of implementation during 2018/19 season.

^b^ Including the nursing home director, physician care coordinator or nursing care coordinator.

^c^ Healthcare worker who could provide reliable information on vaccination.

The numbers of nursing homes and HCWs are provided in Table 1. The difference between the season under review and the 2018/19 season (the most recent pre-COVID season for which estimates are available) was determined by Fisher’s exact test: * p < 0.05 ; ** p < 0.01 ; *** p < 0.001. No comparison was made to the 2007/08 season. Only significant results are reported.

Compared to 2018/19 (the most recent pre-COVID-19 pandemic season for which estimates are available), almost all HCW influenza vaccination measures were significantly less implemented in NHs from 2021/22 onwards. For the majority of measures, the decline continued or the proportion of implementation remained low for all subsequent seasons. For example, in 2024/25 compared with 2018/19, the use of posters (−9 points, p < 0.001), the provision of information on influenza (−14 points, p < 0.001), the organisation of collective information sessions (−19 points, p < 0.001), the provision of information on influenza vaccines (−11 points, p < 0.001) and the organisation of individual information sessions (−10 points, p < 0.001) were significantly less implemented.

Support for the vaccination campaign by NH management teams dropped by 37 percentage points from 2018/19 to 2024/25. From 2021/22 onward, only about half of NHs (52–57%) received management support for the campaign.

Compared to 2018/19 (33%), after sharp declines in 2021/22 (−17 points, p < 0.001) and 2022/23 (−9 points, p < 0.001), the proportion of NHs designating a vaccination contact point rebounded in 2023/24 (31%; 95% CI: 29–34) and 2024/25 (30%; 95% CI: 28–32), nearing 2018/19 levels.

In 2024/25, NHs implementing fewer measures cited these main reasons: team vaccine reluctance (71.4%; 95% CI: 67.1–75.4), staff turnover (26.6%; 95% CI: 22.7–30.7), feeling that actions are not effective (19.1%; 95% CI: 15.7–22.9), lack of time (19.1%; 95% CI: 15.7–22.9), staff shortages (17.0%; 95% CI: 13.8–20.7), low motivation (13.3%; 95% CI: 10.4–16.6) or no care coordinator (2.7%; 95% CI: 1.4–4.6).

### Determinants and measures associated with influenza vaccination coverage for healthcare workers in nursing homes

Independent of season, influenza VC among HCWs was significantly higher in private and private non-profit NHs than in public NHs, and in small NHs (< 100 beds) vs large ones. Influenza VC was significantly lower in NHs affiliated to a hospital than in those that were not (Table 4).

**Table 4:** Determinants of influenza vaccination coverage of healthcare workers in nursing homes, France, 2018/19 (n = 558 nursing homes), 2023/24 (n = 1,561) and 2024/25 (n = 1,681)^a^

Variables	2018/19			2023/24			2024/25		
	PRa	95% CI	p	PRa	95% CI	p	PRa	95% CI	p
**Nursing home characteristics **									
**Affiliated to a hospital**									
No (or ‘I don’t know’)	Ref.			Ref.			Ref.		
Yes	0.80	0.69–0.91	***	0.79	0.73–0.86	***	0.80	0.74–0.87	***
**Sector **									
Public	Ref.			Ref.			Ref.		
Private non-profit	1.05	0.87–1.17	*	1.13	1.04–1.22	**	1.18	1.09–1.28	***
Private	1.28	1.10–1.48	***	1.33	1.20–1.46	***	1.45	1.32–1.59	***
**Size **									
< 100 beds	Ref.			Ref.			Ref.		
≥ 100 beds	0.88	0.82–0.95	***	0.86	0.79–0.95	**	0.84	0.77–0.92	***
**Coordinating physician**									
No	Ref.			NI			NI		
Yes	1.38	1.13–1.69	**						
**Measures implemented **									
**Vaccination free-of-charge for HCWs in the nursing home **									
No	Ref.			NI			Ref.		
Yes	1.43	1.12–1.81	**				1.30	1.10–1.54	**
**Videos or games**									
No	Ref.			NI			NI		
Yes	1.40	1.20–1.64	***						
**Information about influenza vaccines**									
No	Ref.			NI			NI		
Yes	1.16	1.01–1.33	*						
**Information about influenza**									
No	NI			Ref.			Ref.		
Yes				1.20	1.11–1.29	***	1.13	1.05–1.22	**
**Collective information sessions**									
No	Ref.			Ref.			NI		
Yes	1.27	1.09–1.47	**	1.10	1.02–1.19	**			
**Individual information sessions**									
No	Ref.			NI			Ref.		
Yes	1.55	1.13–2.12	**				1.15	1.04–1.28	**
**Point of contact for vaccination nominated within the nursing home^b^**									
No	Ref.			Ref.			Ref.		
Yes	1.69	1.3–2.2	***	1.11	1.03–1.19	**	1.10	1.02–1.18	**
**The director, the care coordinator (physician) or the nursing coordinator are involved and support the vaccination campaign**									
No	Ref.			NI			NI		
Yes	1.25	1.03–1.52	*						

PRa: adjusted multivariate prevalence ratio; Ref.: reference; NI: not included in the multivariate analysis.

^a^ Regions were taken into account in the multivariate analyses. Results are provided in Supplementary Table S6.

^b^ Healthcare worker who could provide reliable information on vaccination.

In the multivariate analysis, significance for the determinants for each season compared to the reference variable are indicated as *p: < 0.05; **: < 0.01; ***: < 0.001.

Concerning measures implemented to support the influenza vaccination campaign, influenza VC among HCWs was significantly higher in 2018/19, when (i) vaccination was provided free-of-charge for HCWs in the NH (+ 43%), when (ii and iii) collective or individuals sessions were organised (+ 27% and + 55%), (iv) when information was provided about influenza vaccines (+ 16%), (v) when videos or games were used (+ 40%), (vi) when a point of contact for vaccination was nominated within the NH (+ 69%) and (vii) when the management team was involved and supported the vaccination campaign (+ 25%).

Higher VC were observed in 2023/24 and in 2024/25 when information was provided about influenza (+ 20% and + 13% respectively) and when a point of contact for vaccination was nominated within the NH (+ 11% and + 10%), in 2023/24 when collective information sessions were organised (+ 10%), and in 2024/25 when vaccination was provided free of charge for HCWs in the NH (+ 30%) and individuals sessions were organised (+ 15%).

In 2018/19, in NHs which provided free-of-charge vaccination, and had a point of contact, and used videos or games and provided information on influenza vaccines, average VC was 53.6% (38.9–68.4) (n = 16 NHs) [10]. In 2023/24 and in 2024/25, in the NHs which provided free-of-charge vaccination, had a point of contact, provided information on influenza, and organised collective information sessions VC were estimated at 32.2% (95% CI: 29.6–34.9) (n = 286 NHs) and 26.1% (95% CI: 22.6–29.5) (n = 96 NHs), respectively.

In contrast, in NHs where none of the significant measures were implemented (no free-of-charge vaccination, and no collective or individuals sessions organised, and no information provided about influenza vaccines, and no videos or serious games used, and no point of contact for vaccination nominated and the management team not involved in the vaccination campaign), the VC of HCWs was lower than 15% in 2018/19, estimated at 18.7% (95% CI: 15.1–22.2) (n = 69 NHs) in 2023/24 and at 17.2% (95% CI: 8.9–25.8) (n = 21 NHs) in 2024/25.

## Discussion

Our national studies conducted between the 2007/08 and 2024/25 seasons in France showed that influenza VC among HCWs working in NHs for older people over 60 years was low and decreased over time. Internationally, VC among HCWs rarely exceeded 30–40% [12], e.g. Germany: 39% in 2017/18 [13] and Cyprus: 32% in 2019/20 [14], but has reached 71% in Qatar in 2013 [15] and nearly 90% in the United States (US) in 2018/19 [16].

Following the emergence of COVID-19, in 2020/21, influenza VC among HCWs working in NHs briefly surged in France (55%), as observed in other countries (Ireland: 71%, Greece: 68%, Portugal: 63%) [17] before declining in subsequent seasons, which mirrored trends in the US [16], Spain [18] and Israel [19]. In France, this high VC may have been accentuated by low NH participation in the 2020/21 season (n = 50 nursing homes). Participation biases cannot be excluded.

Independent of season, influenza VC varied widely depending on the category of HCW. The target of 75% of HCW vaccinated [9] was only achieved for physicians in 2018/19 and 2021/22. Differences in VC across categories of HCWs were also observed for influenza in healthcare facilities in 2019 in France (all HCWs: 35%, physicians: 67%, midwives: 48%, nurses: 36%, nursing assistants: 21%), as well as for measles, pertussis, varicella and COVID-19 [20,21]. Similar patterns have also been observed in Germany [13] and Cyprus [22]. 

The educational levels vary significantly among HCWs categories: physicians hold professional degrees, nurses typically have a bachelor's degree, and nursing assistants may not be required to have a high school diploma. Differences in VC based on education level have already been observed in other populations in France, for example, among pregnant women for influenza [23] and pertussis [24] or among people aged 65 and over for influenza vaccination [25]. 

Our results highlight the importance of investigating the multiple reasons behind the low and declining influenza VC among HCWs. These results suggest that individual factors, e.g. education level, socioeconomic status etc., should be further studied. The expectations or questions regarding vaccination may likely vary depending on professional categories. Support for vaccine decision-making should therefore be tailored to the different categories of HCWs and take into account psychological antecedents of vaccine acceptance (known as the 7C) [26].

The 2018/19 study identified seven measures that could significantly improve influenza VC among HCWs in NHs. When multiple measures were combined, higher VC numbers were observed, but they did not exceed 55% [10]. Since the COVID-19 pandemic, the proportion of NHs implementing these measures significantly decreased. Some measures identified during the 2018/19 season were not effective during the 2023/24 or 2024/25 seasons. In addition, for the measures that were effective during all three seasons, the expected gain in terms of coverage was lower in 2023/24 or 2024/25 than in 2018/19. Two hypotheses could explain these results. The first could be related to the quality of the measures implemented, e.g. clarity of visuals, accessibility, and the involvement of professionals in their implementation, e.g. time devoted, which may have diminished over time. The second could be linked to the effectiveness of the measure itself: with the same levels of quality and professional involvement, a measure that was effective in 2018/19 may no longer be so in 2023/24, because e.g. HCWs are more often reluctant to accept influenza vaccination, or because they experience vaccine fatigue. Our quantitative data do not allow us to assess the quality of the measures implemented in NHs nor to evaluate decreased impact of the measures on professionals’ adherence to influenza vaccination. Our findings suggest that further investigation is needed to understand the factors associated with the declining engagement of HCWs and management teams in the influenza vaccination of HCWs in recent years. It would be advisable to remobilise the management teams of NHs to ensure that proven effective measures are implemented or reintroduced in their facilities.

The main reasons cited by NHs’ management teams to explain the reduction in implemented measures were vaccine reluctance among staff and high turnover rates. The COVID-19 pandemic has undoubtedly exacerbated these challenges. In France, we observed an increase in vaccination acceptance in the general population since the COVID-19 pandemic [27]. These results are not available for HCWs. A focussed study on the acceptance of influenza vaccination among HCWs is needed to determine whether the observed decreases in VC may be associated to a decline in influenza vaccination acceptability among HCWs. The question of mandatory influenza vaccination for HCWs has been raised by the authorities. Mandatory vaccination rapidly achieves very high coverage [28]. However, obstacles to mandatory vaccination include limited vaccine effectiveness, insufficient data on indirect protection for residents [29] and low adherence to mandatory influenza vaccination among HCWs in France (43% in 2019) [30]. It might be useful to test in a study the acceptability and effectiveness of the mandatory declination form (document in which professionals explain in writing why they have not been vaccinated) in France [31] and to mandate influenza vaccination in all NHs for elderly persons in France. 

Our study has limitations. Firstly, VC estimates must take vaccinations into account regardless of where they were administered. As data are self-reported by each NH, it cannot be ruled out that some NH representatives may not be aware of all vaccinations administered outside their NH. Secondly, although no change in the distribution of NH categories was observed across seasons, the existence of selection bias that could influence VC estimates cannot be ruled out. Finally, the data available for the 2020/21 season are partial and should be interpreted with caution. This is due to data collection occurring at a different time of year compared to other seasons, the vaccination campaign not yet being completed, and a significantly lower number of NHs participating in the VC study. It is possible that only those with the highest HCW VC were included, which may introduce bias. 

## Conclusions

Since the COVID-19 pandemic, we observed a substantial decline in national influenza VC among HCWs working in France in NHs. This decrease was observed for almost all categories of HCWs. Measures aimed to promote vaccination of HCWs were less frequently implemented than in the pre-pandemic period, and less effective when implemented. To reverse the decline in influenza vaccination among HCWs, NH management teams must be made aware of the importance of supporting vaccination campaigns by reinstating proven measures.

## Data Availability

Aggregate data are available from the corresponding author at reasonable request.

## Supplementary Data

4460000 Supplement
