# Supplementary material for: Decreases in influenza vaccination coverage among nursing home healthcare workers and in measures to promote influenza vaccination, France, 2007/08 to 2024/25
Source: Euro Surveill. 2026 Mar 26;31(12):2500628. doi: 10.2807/1560-7917.ES.2026.31.12.2500628 (PMC13074129; doi:10.2807/1560-7917.ES.2026.31.12.2500628)
Supplement: Supplement [file 25-00628_VAUX_Supplement.pdf]

SUPPLEMENTARY MATERIAL

This supplementary material is hosted by Eurosurveillance as supporting information alongside the article “Decreases in influenza vaccination coverage among nursing homes’ healthcare workers and in measures implemented to promote influenza vaccination, France, 2007/08 to 2024/25”, on behalf of the authors, who remain responsible for the accuracy and appropriateness of the content. The same standards for ethics, copyright, attributions and permissions as for the article apply. Supplements are not edited by Eurosurveillance and the journal is not responsible for the maintenance of any links or email addresses provided therein.

SUPPLEMENT S1: Questionnaire of VC studies, translate in English: pages 1-3

SUPPLEMENT S2: Numbers of nursing homes and participation of nursing homes included in the study by region, France 2007/08 - 2024/25 (6 seasons): page 4

SUPPLEMENT S3: Influenza vaccination coverage for HCWs in nursing homes, France, from 2007/08 to 2024/25 seasons: page 5

SUPPLEMENT S4: Regional vaccine coverage for influenza for HCWs in nursing homes, France, 2018/19 and 2024/25 seasons: pages 6 - 7

SUPPLEMENT S5: Measures implemented in nursing homes for HCWs influenza vaccination, France, from 2007/08 to 2024/25 seasons: page 8

SUPPLEMENT S6. Determinants of influenza vaccination coverage of healthcare workers in nursing homes, France: pages 9-11

SUPPLEMENT S1: Questionnaire of VC studies, translate in English

Monitoring influenza vaccination coverage among Healthcare Workers in nursing homes for elderly

Questionnaire – 2024-2025 season

Administrative Data

Name of the nursing home: _

Geographic FINESS number of the nursing home: /____/____/____/____/____/____/____/____/____/

Departement of the nursing home: __

Date on which you are completing this questionnaire: /____/____/____/

Affiliation with a healthcare facility: (i.e., the institution shares the same management as a healthcare facility)

☐ Yes ☐ No ☐ Unknown

Influenza vaccination of professionals

For nursing homes located in the Southern Hemisphere: consider vaccinations administered during the 2024 austral winter.

	**Number of salaried professionalsin the nursing home** 1)	**Number of professionals vaccinated against influenza since October 15, 2024** 1) 2)
Physicians or pharmacists		_ _ _ _ ☐ I don't know
Nurses, including specialized nurses		_ _ _ _ ☐ I don't know
Nursing assistants		_ _ _ _ ☐ I don't know
Other paramedical staff, rehabilitation personnel, psychologists		_ _ _ _ ☐ I don't know

1) Salaried professionals employed at the nursing home during the last season (since October 2024).
Do not calculate in full-time equivalent (FTE), but report the actual number of individuals.
Do not include temporary staff present for less than 3 months, nor self-employed professionals (e.g., independent practitioners) working at the facility.
Only fill in the categories where there are more than 2 relevant staff members.

2) Professionals vaccinated either within or outside the nursing home. Indicate the number of professionals for whom you have confirmed influenza vaccination for the 2023-2024 season. Check "Do not know" if you have no information. Note: You cannot enter a number and check "Do not know" at the same time

Do the numbers of professionals vaccinated against influenza include vaccinations administered outside your facility

☐ Yes, completely ☐ Yes, partially ☐ No, not at all ☐ I don’t know

What actions were implemented this season (2024-2025) in your nursing home to promote influenza vaccination among professionals? (Multiple answers possible)

☐ Organization of vaccination for professionals by the occupational health service

☐ Organization of free vaccination for professionals within the nursing home

☐ Posters

☐ Videos, serious games

☐ Information about influenza vaccines

☐ Information about influenza

☐ Collective information sessions

☐ Individual information sessions

☐ Point of contact for vaccination nominated within the nursing home (e.g., physicians or paramedical staff who can provide reliable information on vaccination)

☐ Existence of a multidisciplinary group on vaccination in the nursing home (e.g., a group including physicians, nurses, nursing managers, occupational health physicians, hygiene teams, or infectious disease specialists to define campaign organisation and actions to improve vaccination coverage)

☐ The director, the care coordinator (physician) or the nursing coordinator are involved and support the vaccination campaign

☐ Other: please specify : ________________________________

The number of actions implemented to promote influenza vaccination among professionals in your facility during the 2024-2025 season was:

☐ Greater than in previous years*

☐ Equivalent to previous years*

☐ Less than in previous years*

☐ I don’t know

*Ideally, compared to years before the COVID-19 pandemic

If the number of actions implemented this season was lower than in previous years, or if very few actions were implemented, what were the reasons? (Multiple answers possible)

☐ Staff shortages

☐ High team turnover

☐ Lack of motivation

☐ Strong vaccine hesitancy among staff

☐ Perception that actions are ineffective

☐ Less time available

☐ Other: please specify : ________________________________

TABLE S2. Numbers of nursing homes and participation of nursing homes included in the study by region, France 2007/08 - 2024/25 (6 seasons)[a]

	Participation per season											
	Numbers of nursing homes included						Participation (%)					
Season	2007/08[b]	2018/19[c]	2021/22	2022/23	2023/24	2024/25	2007/08 [b]	2018/19[c]	2021/22	2022/23	2023/24	2024/25
Auvergne-Rhône-Alpes	NA	78	136	297	327	601	NA	61.4	14.6	31.9	35.2	44.4
Bourgogne-Franche-Comté	NA	42	68	110	153	305	NA	54.5	16.3	26.4	37.2	51.7
Bretagne	NA	45	60	78	112	174	NA	57.7	11.7	15.2	21.8	26.8
Centre-Val de Loire	NA	36	59	79	84	133	NA	45.0	18.8	25.2	26.8	30.7
Corse	NA	26	2	6	10	2	NA	92.9	6.9	20.7	34.5	3.4
Grand Est	NA	40	109	155	117	296	NA	48.8	17.9	25.4	19.2	33.3
Hauts-de-France	NA	38	107	104	104	238	NA	48.7	18.3	17.8	18.1	31.0
Ile-de-France	79	17	225	376	214	306	41.9	17.9	31.8	53.1	30.2	28.1
Normandie	NA	38	80	82	161	218	NA	47.5	21.0	21.5	42.3	48.0
Nouvelle-Aquitaine	NA	59	208	321	372	408	NA	47.2	22.7	35.0	40.9	30.9
Occitanie	NA	44	124	187	268	509	NA	39.6	15.1	22.7	32.7	41.9
Pays de la Loire	NA	49	78	63	169	224	NA	62.0	13.4	10.8	29.1	27.5
Provence-Alpes-Côte d'Azur	NA	40	166	143	263	287	NA	50.0	28.0	24.1	44.4	34.9
Guadeloupe	NI	9	0	1	0	17	NI	42.9	0	4.5	0	59.1
Guyane	NI	3	0	0	3	2	NI	75.0	0	0	60.0	20.0
La Réunion	NI	12	9	3	10	14	NI	48.0	42.9	14.3	50.0	40.0
Martinique	NI	12	0	1	3	1	NI	63.2	0	4.0	12.0	4.0
France	1218	589	1431	2006	2370	2630	55.7	49.5	19.1	26.9	31.9	35.5

NA: not available; NI: not included in the study

[a] During the 2020/21 season, the number of participating nursing homes was 256 (vaccination for HCWs was complete for only 50 nursing homes).

[b] For the 2007/08, participation of nursing homes are not available by region

Figure S3. Influenza vaccination coverage for HCWs in nursing homes, France, from 2007/08 to 2024/25 seasons

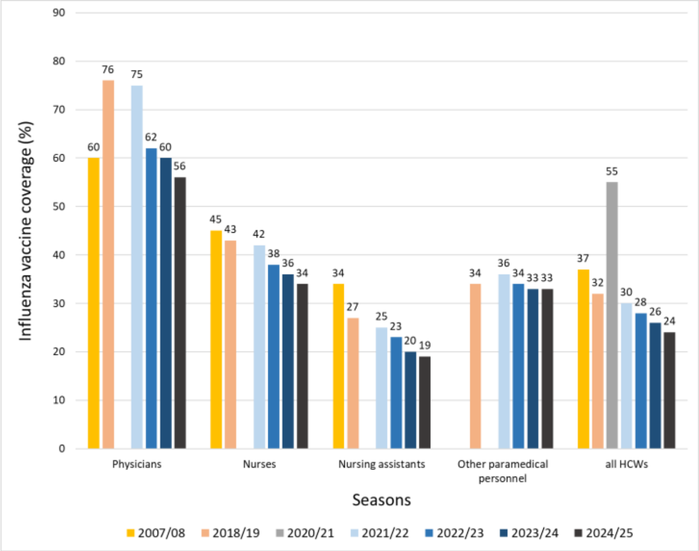

Figure S4. Regional vaccine coverage for influenza for HCWs in nursing homes, France, 2018/19 and 2024/25 seasons

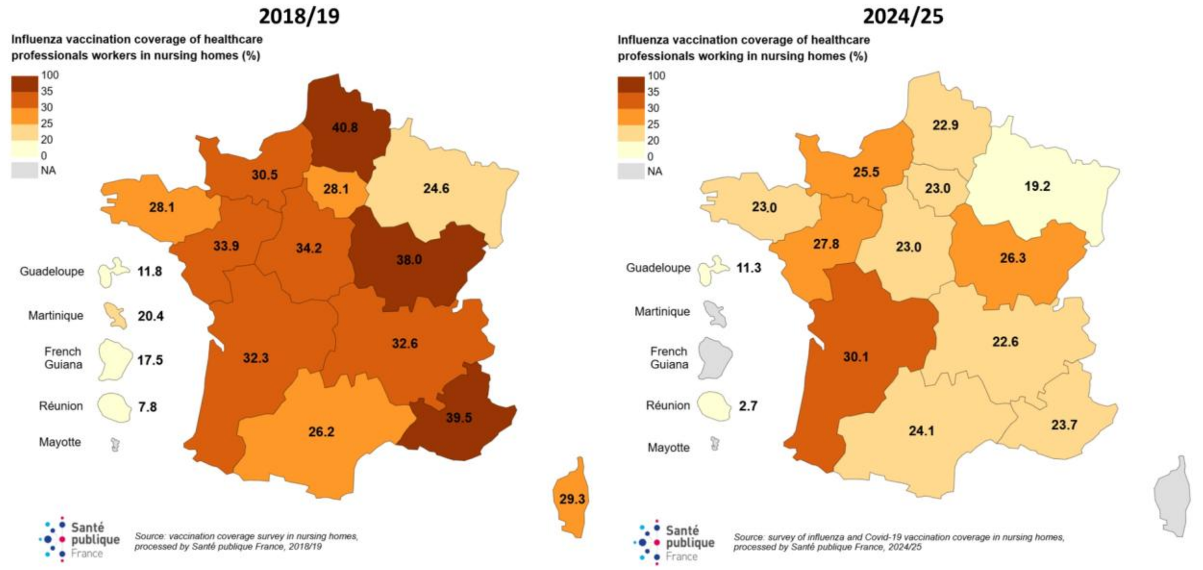

More regional data available:

2007-2008 season: Vaux S, Noël D, Fonteneau L, Guthmann JP, Lévy-Bruhl D. Influenza vaccination coverage of healthcare workers and residents and their determinants in nursing homes for elderly people in France: A cross-sectional survey. BMC Public Health. 2010;10. https://bmcpublichealth.biomedcentral.com/articles/10.1186/1471-2458-10-159

2018-2019 season: Vaux S, Fonteneau L, Venier AG, Gautier A, Soing Altrach S, Parneix P, et al. Influenza vaccination coverage of professionals working in nursing homes in France and related determinants, 2018–2019 season: a cross-sectional survey. BMC Public Health. 2022;22(1). https://bmcpublichealth.biomedcentral.com/articles/10.1186/s12889-022-13412-5

Vaux S, Fonteneau L, Lévy-Bruhl D. Couvertures vaccinales des professionnels de santé contre la grippe. Bulletin de Santé publique Vaccination. Edition nationale. Octobre 2019. Saint-Maurice : Santé publique France https://www.santepubliquefrance.fr/determinants-de-sante/vaccination/documents/bulletin-national/bulletin-de-sante-publique-vaccination.-octobre-2019

2020-2021 season: Santé publique France. Etude de couverture vaccinale contre la grippe chez les professionnels de santé et les résidents des Ehpad, saison 2020-2021. Saint-Maurice : Santé publique France https://www.santepubliquefrance.fr/determinants-de-sante/vaccination/articles/etude-de-couverture-vaccinale-contre-la-grippe-chez-les-professionnels-de-sante-et-les-residents-des-ehpad-saison-2020-2021

2021-2022 season: Santé publique France. Quelle est la couverture vaccinale contre la grippe des résidents et des professionnels exerçant en établissements médico-sociaux. Saison 2021-2022. Le point sur, 1er juin 2022. Saint-Maurice : Santé publique France. https://www.santepubliquefrance.fr/etudes-et-enquetes/etude-de-couverture-vaccinale-contre-la-grippe-et-la-covid-19-dans-les-etablissements-medico-sociaux-et-etablissements-de-sante-saison-2021-2022

2022-2023 season: Santé publique France. Quelle est la couverture vaccinale contre la grippe des résidents et des professionnels en établissements sociaux et médico-sociaux (ESMS). Saison 2022-2023. Le point sur, Juillet 2023. Saint-Maurice : Santé publique France : https://www.santepubliquefrance.fr/etudes-et-enquetes/etude-de-couverture-vaccinale-contre-la-grippe-des-residents-et-professionnels-salaries-des-etablissements-medico-sociaux-2023

2023-2024 season: Couvertures vaccinales contre la grippe et la Covid-19 des résidents et contre la grippe des professionnels en établissements sociaux et médico-sociaux (ESMS). Le point sur, juillet 2024. Saint-Maurice : Santé publique France, 13 p. https://www.santepubliquefrance.fr/maladies-et-traumatismes/maladies-et-infections-respiratoires/grippe/documents/enquetes-etudes/couvertures-vaccinales-contre-la-grippe-et-la-covid-19-des-residents-et-contre-la-grippe-des-professionnels-en-etablissements-sociaux-et-medico-soc

2024-25 season: Couvertures vaccinales contre la grippe et la Covid-19 des résidents et des professionnels en établissements sociaux et médico-sociaux (ESMS). Le point sur, juillet 2025. Saint-Maurice : Santé publique France, 16 p. https://www.santepubliquefrance.fr/maladies-et-traumatismes/maladies-et-infections-respiratoires/grippe/documents/enquetes-etudes/couvertures-vaccinales-contre-la-grippe-et-la-covid-19-des-residents-et-des-professionnels-en-etablissements-sociaux-et-medico-sociaux-esms-.-poin

Figure S5. Measures implemented in nursing homes for HCWs influenza vaccination, France, from 2007/08 to 2024/25 seasons

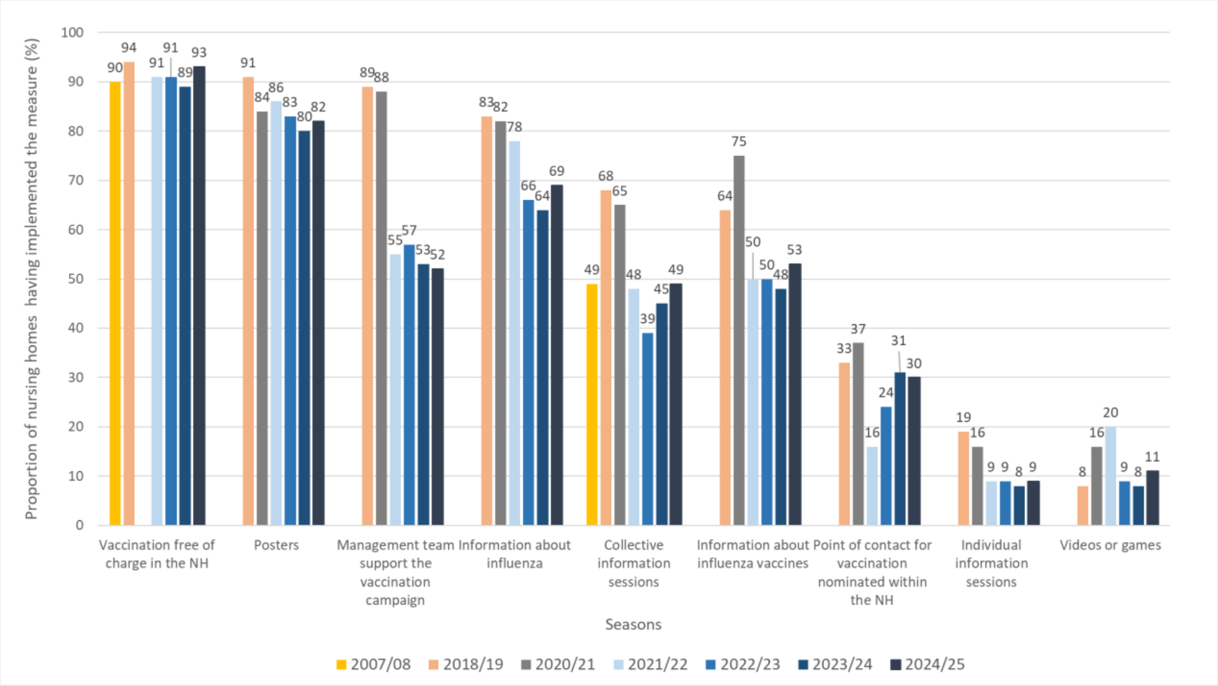

TABLE S6. Determinants of influenza vaccination coverage of healthcare workers in nursing homes, France, 2018/19 (n = 558 nursing homes), 2023/24 (n = 1,561) and 2024/25 (n = 1,681)

	2018-2019			2023-2024			2024-2025		
**Variables**	Nb of NHs	PRa [95%CI]	p	Nb of HCWs	PRa [95%CI]	p	Nb of HCWs	PRa [95%CI]	p
**Nursing home characteristics**									
**Affiliated to a hospital**									
No (or 'I don't know')	366	ref		32,960	ref		37,046	ref	
Yes	159	0.80 [0.69-0.91]	***	21,464	0.79 [0.73-0.86]	***	20,625	0.80 [0.74-0.87]	***
**Sector**									
Public	251	ref		31,264	ref		33,266	ref	
Private non-profit	119	1.05 [0.87-1.17]	*	13,686	1.13 [1.04-1.22]	**	15,020	1.18 [1.09-1.28]	***
Private	176	1.28 [1.10-1.48]	***	8,069	1.33 [1.20-1.46]	***	8,440	1.45 [1.32-1.59]	***
**Size**									
< 100 beds	109	ref		35,362	ref		37,207	ref	
≥ 100 beds	436	0.88 [0.82-0.95]	***	12,916	0.86 [0.79-0.95]	**	17,235	0.84 [0.77-0.92]	***
**Coordinating physician**									
No	80	ref		NI	NI		NI	NI	
Yes	461	1.38 [1.13-1.69]	**	NI	NI		NI	NI	
**Measures implemented**									
**Vaccination free of charge for HCWs in the nursing home**									
No	17	ref		NI	NI		12,778	ref	
Yes	531	1.43 [1.12-1.81]	**	NI	NI		44,893	1.30 [1.10-1.54]	**
**Posters**									
No	NI	NI		NI	NI		NI	NI	
Yes	NI	NI		NI	NI		NI	NI	
**Videos or serious games**									
No	496	ref		NI	NI		NI	NI	
Yes	44	1.40 [1.20-1.64]	***	NI	NI		NI	NI	
**Information about influenza vaccines**									
No	190	ref		NI	NI		NI	NI	
Yes	350	1.16 [101-1.33]	*	NI	NI		NI	NI	

**Information about influenza**									
No	NI	NI		19,591	ref		16,548	ref	
**Yes**	NI	NI		34,833	1.20 [1.11-1.29]	***	41,123	1.13 [1.05-1.22]	**
**Collective information sessions**									
No	184	ref		29,655	ref		NI	NI	
**Yes**	356	1.27 [1.09-1.47]	**	24,769	1.10 [1.02-1.19]	**	NI	NI	
**Individual information sessions**									
No	431			NI	NI		51,389	ref	
Yes	109	1.55 [1.13-2.12]	**	NI	NI		6,282	1.15 [1.04-1.28]	**
**Point of contact for vaccination nominated within the nursing home [a]**									
No	370	ref		33,343	ref		38,338	ref	
**Yes**	170	1.69 [1.30-2.20]	***	21,081	1.11 [1.03-1.19]	**	19,333	1.10 [1.02-1.18]	**
**In-house multidisciplinary group on vaccination**									
No	NI	NI		NI	NI		NI	NI	
Yes	NI	NI		NI	NI		NI	NI	
**The director, the care coordinator (physician) or the nursing coordinator are involved and support the vaccination campaign**									
No	62	ref		NI	NI		NI	NI	
Yes	486	1.25 [1.03-1.52]	*	NI	NI		NI	NI	
**Region**									
Hauts-de-France	NI	NI		3,795	ref		4,812	1.01 [0.87-1.16]	
Auvergne-Rhône-Alpes	NI	NI		8,838	0.91 [0.77-1.08]		10,133	1.02 [0.90-1.15]	
Bourgogne-Franche-Comté	NI	NI		3,711	1.10 [0.91-1.34]		5,161	1.23 [1.07-1.42]	**
Bretagne	NI	NI		2,873	0.96 [0.78-1.18]		2,505	1.11 [0.93-1.33]	
Centre-Val de Loire	NI	NI		2,539	0.94 [0.74-1.19]		2,808	1.23 [1.02-1.48]	*
Grand Est	NI	NI		3,598	0.91 [0.74-1.13]		4,782	0.89 [0.77-1.03]	
Ile-de-France	NI	NI		3,367	0.96 [0.79-1.16]		3,391	0.98 [0.83-1.15]	
Normandie	NI	NI		3,446	1.03 [0.85-1.25]		4,593	1.15 [0.99-1.33]	
Nouvelle-Aquitaine	NI	NI		7,361	1.05 [0.89-1.24]		5,454	1.22 [1.07-1.40]	**
Occitanie	NI	NI		6,010	0.88 [0.74-1.05]		6,706	ref	
Pays de la Loire	NI	NI		3,034	1.11 [0.92-1.34]		2,733	1.32 [1.11-1.57]	***
Provence-Alpes-Côte d'Azur	NI	NI		5,806	0.72 [0.60-0.86]	**	3,989	0.89 [0.76-1.03]	

La Réunion	NI	NI		NI	NI		224	0.12 [0.05-0.31]	***

NI: not included in the multivariable analysis

[b] Healthcare worker who can provide reliable information on vaccination.
